# Erythropoietin (EPO) as a Key Regulator of Erythropoiesis, Bone Remodeling and Endothelial Transdifferentiation of Multipotent Mesenchymal Stem Cells (MSCs): Implications in Regenerative Medicine

**DOI:** 10.3390/cells10082140

**Published:** 2021-08-20

**Authors:** Asterios S. Tsiftsoglou

**Affiliations:** Laboratory of Pharmacology, Department of Pharmaceutical Sciences, Aristotle University of Thessaloniki, 54124 Thessaloniki, Greece; tsif@pharm.auth.gr

**Keywords:** erythropoietin-alpha, bone marrow erythropoiesis, bone remodeling, endothelial transdifferentiation (angiogenesis), mesenchymal stem cells, regenerative medicine

## Abstract

Human erythropoietin (EPO) is an N-linked glycoprotein consisting of 166 aa that is produced in the kidney during the adult life and acts both as a peptide hormone and hematopoietic growth factor (HGF), stimulating bone marrow erythropoiesis. EPO production is activated by hypoxia and is regulated via an oxygen-sensitive feedback loop. EPO acts via its homodimeric erythropoietin receptor (EPO-R) that increases cell survival and drives the terminal erythroid maturation of progenitors BFU-Es and CFU-Es to billions of mature RBCs. This pathway involves the activation of multiple erythroid transcription factors, such as GATA1, FOG1, TAL-1, EKLF and BCL11A, and leads to the overexpression of genes encoding enzymes involved in heme biosynthesis and the production of hemoglobin. The detection of a heterodimeric complex of EPO-R (consisting of one EPO-R chain and the CSF2RB β-chain, CD131) in several tissues (brain, heart, skeletal muscle) explains the EPO pleotropic action as a protection factor for several cells, including the multipotent MSCs as well as cells modulating the innate and adaptive immunity arms. EPO induces the osteogenic and endothelial transdifferentiation of the multipotent MSCs via the activation of EPO-R signaling pathways, leading to bone remodeling, induction of angiogenesis and secretion of a large number of trophic factors (secretome). These diversely unique properties of EPO, taken together with its clinical use to treat anemias associated with chronic renal failure and other blood disorders, make it a valuable biologic agent in regenerative medicine for the treatment/cure of tissue de-regeneration disorders.

## 1. Introduction into Human Erythropoietin (EPO): Brief History and Structure

**Figure 1:**
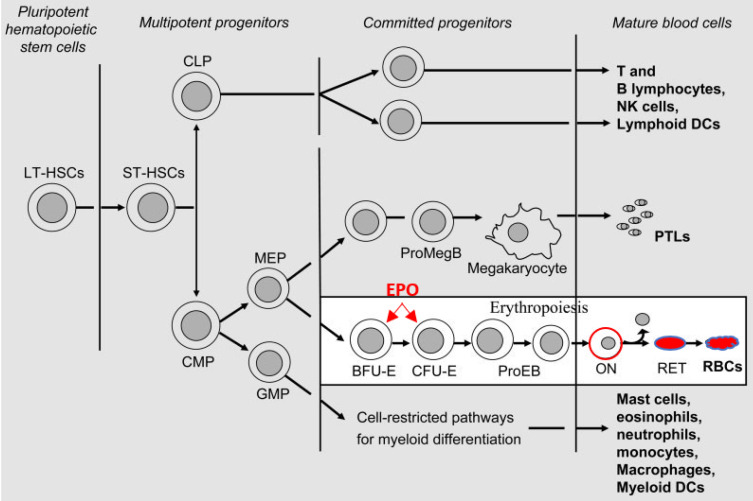
Illustration of the human bone marrow cell restricted pathways of the differentiation of hematopoietic stem cells into mature blood cells. In the lower right box, the erythroid cell restricted lineage differentiation is shown. HSCs give rise to common myeloid progenitors (CMP) and common megakaryocyte erythroid progenitors (MEP). The latter are differentiated into erythropoietin-responsive BFU-E and CFU-E clones, shown by the arrows where human EPO acts upon them. Interactions of EPO with its homodimeric EPO-R lead to cell signaling activation pathways that increase the biosynthesis of hemoglobin. The levels of hemoglobins increase as erythropoiesis continues in the proerythroblasts, in the orthochomatic normoblasts (ON) and finally in the reticulocytes and mature red blood cells (RBCs) following the extrusion of the nucleus. Erythropoiesis is homeostatically regulated by low-oxygen conditions (hypoxia) and the final end production via a feedback action loop. The original figure was published by Tsiftsoglou et al. [4] and has been lightly modified accordingly to indicate where EPO acts upon in the proerythroid cells. The initial commitment to the erythroid cell lineage restricted pathway of differentiation begins at the level of BM HSCs and leads to the formation of MEPs that yield BFU-Es and CFU-Es. The most recently published work by Eisele et al. [9] provided direct evidence indicating that EPO indeed acts upon the BM HSCs to promote commitment towards the erythroid maturation.

Human erythropoietin (EPO) is a peptide hormone produced in the fetal liver during early development and the kidneys in the human adult body. As a major hematopoietic growth factor (HGF), it regulates bone marrow erythropoiesis by promoting the daily massive production (200 billion) of RBCs full of hemoglobin A (α_2_β_2_), that carry oxygen and transfer it from the lungs to tissues [1,2,3,4,5,6] (Figure 1). In terms of structure, EPO is a glycoprotein consisting initially of 193 aa that include an N-terminal 27 aa signaling peptide. This premature polypeptide is post-translationally cleaved to yield the mature form of EPO (UniProt P01588).

The primary structure of the full-length human erythropoietin contains 166 aa, with an approximate MW of 30.4 kDa. It is an acidic glycoprotein and exhibits two disulfide (-S-S-) bonds, supporting the overall molecular structure between Cys29 and Cyst33 and Cys7 and Cys161. There are three N-linked carbohydrates at the Asparagine residues 24, 38 and 83, and one O-linked carbohydrate at Serine 126. The 3D structure of EPO is composed of four alpha-helices. Helix A is connected to Helix D through Cys7 and Cys171, while Helices A and B are connected through Cys29 and Cys33. Over the years, various recombinant human erythropoietins (rhEPOetins) have been synthesized in several gene-cloning-expression platforms. These include genetically modified mammalian CHO [7,8] and HEK293 cells, as well as yeast- and baculovirus-infected insect cell systems. The biosimilar epoetins exhibit some differences attributed to variations in the glycosylation patterns. rhEPO has been commercially available since 1989 after gaining authorization approval by the FDA (EPOGEN^R^) for use as a valuable biologic medicinal product in the replacement protein therapy (RPT) of chronic renal failure (CRF), that is characterized by insufficient production of EPO.

Historically, the search for EPO began as early as 1875, when anemia-like symptoms were diagnosed in patients living at low altitude and characterized by low blood oxygen levels. Moreover, it took intensive research work over the past 50 years to identify EPO as the major driver of BM erythropoiesis, purify it from urine derived from patients with aplastic anemia by Goldweisser’s group, clone the EPO gene and produce recombinant EPO, as mentioned earlier [7,8,10,11,12,13]. The elegant work of the Lodish group at MIT in 1989 [14] led to the discovery of the EPO-receptor (EPO-R), which provoked further studies on the EPO–EPO-R interactions and downstream signaling processes.

This comprehensive review aims to cover the pleiotropic activity of EPO as a major growth factor/promoter of erythropoiesis, an activator of BM bone remodeling as well as an inducer of endothelial transdifferentiation/angiogenesis of multipotent mesenchymal stem cells (MSCs) into different cell types. The latter includes osteoblasts, odontoblasts, chondrocytes, endothelial cells and others. Moreover, this review will present the mechanisms of EPO production via an oxygen-sensitive feedback loop, as well as its potential to activate the interactions of EPO–EPO-R and provoke cell signaling pathways involved in the modulation of immune cell functions as well as angiogenesis via the endothelial differentiation of MSCs. Last but not least, the use of EPO and its biosimilars used clinically for different indications will be updated. Unfortunately, the very interesting functions of EPO as a survival and protection factor for other tissues, including the brain (CNS) and heart, as well as on fat accumulation and obesity revealed over the recent years, will not be presented. Reviews covering these topics are available [15,16].

## 2. Homeostatic Regulation of EPO Production via an Oxygen-Sensitive Feedback Loop

An elaborate oxygen-sensing molecular mechanism was discovered in the early 1990s [3,4,14,17] to explain the regulation of EPO production under hypoxic conditions. Under ischemic stress, anemia, hypoxia at high altitude as well as other factors (tissue injuries, degeneration), the production of EPO is activated in the adult kidneys. EPO is secreted into plasma and stimulates the BM erythroid progenitors via the EPO-R to undergo erythroid maturation into RBCs [3,4,5,6,16,18]. However, as the EPO-R is not restricted exclusively on erythroid cells, but is also expressed on several other tissues (heart, bone, brain neurons, adipose tissue, endothelial cells, immune cells), the biological effects of EPO on other tissues makes this hormone (or otherwise defined as hematopoietic growth factor) a very interesting pleiotropic macromolecule (see Table 1).

The interstitial peritubular cells (IPCs) of the kidney have been found to produce and secrete EPO during the adult life. Under hypoxia, an increasing number of IPCs produce and secrete EPO in the plasma. Although much less EPO is produced in other tissues, this does not substitute that secreted by the kidney. EPO is a hypoxia-inducible hormone, regulated by the HIF transcription factor that consists of a heterodimer (HIF1α/HIF1β) that binds to a hypoxia response element in the promoter of the EPO gene. Under normoxia (normal oxygen availability), the oxygen-dependent proline hydroxylases downregulate the HIF1α stability/activity, as the hydroxylated HIF1α is marked by ubiquitination by the pVHL complex and degraded in the 26S proteasome [3,4,5,16]. Alternatively, under hypoxia (reduced oxygen availability), HIF1α is structurally stabilized and increased in the renal EPO-producing cells, where it binds on the promoter region of the EPO gene and transactivates it. An increase in the EPO end product then leads to a feedback loop inhibition of the HIF1α transactivation via blockade of interactions with co-activator proteins. Briefly, the hypoxia pathway proteins are master regulators of erythropoiesis [19]. The discovery of the hypoxia-inducing factor (HIF) as the major transactivator of the EPO gene expression mechanistically explained the regulation of EPO synthesis and paved the groundwork for the award of the Nobel prize in Physiology or Medicine to William G. Kaelin, Jr., Sir Peter John Ratcliffe and Gregg L. Semenza in 2019 [20].

**Table 1:** The pleiotropic biological functions of erythropoietin (EPO) on human bone marrow erythroid progenitors and MSCs derived from non-hematopoietic tissues.

1. Bone marrow erythropoiesis via stimulation of proliferation, survival and differentiation of erythroid progenitors into mature red blood cells (RBCs) [3,4,5,18,21]
2. Bone remodeling by activation of the osteoblast bone-forming activity [22,23,24,25,26,27]
3. Modulation of innate and adaptive immunity and inhibition of inflammation [28,29,30,31]
4. Angiogenesis via endothelial transdifferentiation of mesenchymal stem cells (MSCs) [27,32,33,34,35,36,37,38,39]
5. Wound-healing from skeletal muscle injuries via differentiation of muscle progenitors and MSCs [40,41,42,43,44,45]
6. Protection from heart ischemia by increasing the survival of heart cells and endothelial transdifferentiation (angiogenesis) of MSCs [32,41,46,47,48,49,50]
7. Brain protection from ischemic injuries and degenerative disorders via protection of neurons [14,16,51,52,53,54]
8. Inhibition of adipogenesis, fat accumulation and obesity [26]
9. Regulation of energy metabolism via the activation of mitochondrial bioenergetics [21,26,55,56]

## 3. EPO Binds to EPO-R in BM Hematopoietic Progenitors and Promotes Erythroid Differentiation

**Figure 2:**
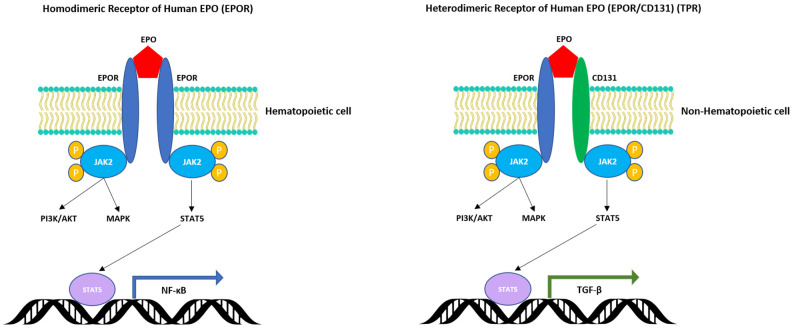
Illustration of the interactions of human EPO with its homodimeric (EPO-R) and heterodimeric receptor (EPO-R/CD131, or tissue protection receptor (TPR)). These receptors are activated in hematopoietic (erythroid progenitors) and non-hematopoietic cells (brain, heart, skeletal muscle cells, others), respectively. Activation of the homodimeric EPO-R receptor leads to cell signaling in BM proerythroid cells, while activation of the heterodimeric EPO-R/CD131 leads to the increase of cell renewal, survival and protection of non-hematopoietic cells. For both cases of EPO-R signaling, the activation pathways shown above are mediated by the phosphorylated JAK2 kinases, which then interact with the intracellular regions of the receptor and activate the PI3K/AKT, MAPK and STAT5 molecules by a phosphorylation cascade. The transcription regulator STAT5 interacts with consensus DNA sequences that facilitate the activation of the NF-kB transcription factor [5,6,57]. In the case of the activation of the EPO-R/CD131 receptor by EPO in non-hematopoietic tissues, the induced cell signaling promotes the phosphorylation of similar molecules, the activation of STAT5 and eventually the expression of TGF-β [28].

ERO-R is a protein cell surface receptor chain encoded by the EPOR gene [3,13]. It carries a single N-linked carbohydrate chain that contributes to an overall approximate molecular weight of 56–57 kDa. ERO-R is a member of the type I cytokine receptor superfamily. As a single transmembrane protein, it contains three structural domains: (i) a 226 aa extracellular domain involved in the binding of EPO, (ii) a 23 aa transmembrane helical domain that facilitates the critical intracellular transfer of any extracellular EPO-induced conformational changes and (iii) a 235 aa C-terminal intracellular domain that contains JAK2 binding sites and 8-tyrosine phosphorylation sites that serve as binding sites for transcription factors such as STAT5, which activate downstream processes [5]. It usually exists as a homodimer and undergoes the conformational changes mentioned that are attributed to the autophosphorylation of the JAK2 kinase associated with the receptor (Figure 2). EPO-R is expressed extensively in EPO-responsive BM erythroid progenitors (BFU-E and CFU-E) derived from the myeloid-erythroid and megakaryocyte (MEK) common precursor, as well in other tissues to certain various levels [3,4,18]. The interaction of EPO with EPO-R at the level of proerythroid cells activates proliferation, increases survival and promotes cell-restricted lineage erythroid differentiation. The EPO-induced cells are committed to the erythroid pathway irreversibly through the sequential intra-conversion to pro-erythroblasts (ProE, BasoE, PolyE), before becoming fully differentiated into orthochromatic erythroblasts (normoblasts). The latter then extrude out of their nucleus to transform into reticulocytes that eventually mature to become red blood cells (RBCs) (Figure 1). This track of hematopoietic cell differentiation has been well-studied in several model systems [4]. The binding of EPO to EPO-R leads to stimulation of the cell signaling pathway, which activates the major erythroid transcription factors GATA-1 and TAL-1, that in turn transactivate the EPOR gene expression. Furthermore, EPO induces heme biosynthesis in the mitochondria and activates the transferrin-mediated iron transport via the cell surface transferrin receptor cycling complex in erythroid progenitors. Binding of EPO to the homodimeric EPOR forms a complex that activates the program of BM erythropoiesis [3,4,5,6]. As the erythroid differentiation proceeds to completion, the EPOR gene expression is transcriptionally downregulated. Recently, detailed investigations of the EPO–EPO-R-induced signaling pathways in disease states have begun to reveal several inherited and acquired disorders associated with either deficient or excessive production of RBCs, as reviewed elsewhere [5]. Therefore, novel “designer” biopharmaceuticals are necessary to control such abnormalities of erythropoiesis.

## 4. EPO Promotes Bone Remodeling

EPO-R receptors have been detected on several other cell types and tissues in addition to the BM erythroid progenitors [21]. These include, among others, heart and neuron cells [46,58], BMMSCs (bone marrow mesenchymal stromal cells) [40], skeletal muscle cells [22] as well as others such as dental MSCs [23,59]. The MSCs are multipotent stem cells capable of undergoing differentiation to osteoblasts, osteoclasts, chondrocytes, odontoblasts, adipocytes and endothelia, according to the stimuli conditions in their microenvironment [24,60,61,62,63,64,65,66,67,68,69]. EPOR is expressed in bone-forming osteoblasts and osteoclasts involved in the bone renewal and resorption respectively, and very important in the regulation of bone homeostasis. Several in vitro and in vivo biological model systems have so far been employed to investigate whether EPO promotes bone remodeling, as recently reviewed by Suresh et al. [6]. In vitro culture studies with BM MSCs provided evidence that EPO increases the osteoblastic, but not the osteoclastic activity [25]. Interestingly, studies with primary osteogenic and adherent BMMSCs have shown that the ability of EPO to increase osteoblastic differentiation is dose-dependent (low: 5 IU/mL vs. high: up to 250 IU/mL). Moreover, in patients suffering from myelodysplastic syndromes (MDS), the effect of EPO was also age-dependent. In a model of fracture repair, a high dose of EPO promoted endochondrial ossification and bone mineralization. In addition, it has also been shown that EPO promoted bone remodeling and endosteal vascularization. In studies designed to use EPO to recruit BMMSCs at sites of bone defects in rats, i.m. injection of EPO mobilized BMMSCs for modeling/repair [25]. Furthermore, injection of EPO at a high dose (4000 IU/dose) for 2 weeks after surgery, in patients with tibiofibular fracture, sped up bone repair by accelerating bone healing. The transplantation of BMMSCs in immunosuppressed animals and the parallel monitoring of bones’ possible formation has shown that bone formation depends on the EPO–EPO-R signaling to affect ectopic bone formation. Based on the summary of the studies discussed, further investigation is needed to pharmacologically optimize the dose of EPO necessary for promoting bone remodeling and the maintenance of a regenerative bone homeostasis (see review) [6,26].

## 5. EPO Induces Angiogenic Differentiation of Human Endothelial Cells (ECs) and Mesenchymal Stem Cells (MSCs)

Earlier studies showing that EPOR is expressed in human umbilical vein endothelial cells (known as HUVEC cells), used as a suitable model system for in vitro angiogenesis of endothelial cells (ECs), have indicated that EPO induces signaling that increases STAT5 phosphorylation and the downstream transcriptional activation of several genes involved in proliferation, migration and capillary vessel formation [32,33]. Pioneering work by Anagnostou et al. in the 1990s confirmed that EPO has a mitogenic and chemotactic effect on cultured HUVEC cells in vitro, and that ECs express EPOR mRNA [34,70]. Further studies generated evidence that EPO stimulates the endothelial progenitor cell mobilization and demonstrated that EPO induces a pro-angiogenic phenotype in cultured ECs and eventually promotes new vascularization in vivo [35,71].

EPO and hypoxia have been shown to stimulate EPOR-mediated vascularization and increased production of nitric oxide (NO) in ECs [72]. This was not induced by the endothelin 1 produced by ECs, but rather through the activation of the signaling pathway AKT-e-Nos-MMP2 by the EPO–EPOR interactions. Production of NO affects the vascular tone of the vascular bed. These angiogenic effects of human EPO in ECs, in terms of ischemic heart and retinal neovascularization, as well as tumor angiogenesis and other pathologies, have already been reviewed [33]. Additional studies by Wan et al. [27] and Holstein et al. [36] demonstrated that EPO promotes chondrogenesis and angiogenesis during bone repair and remodeling, as mentioned earlier, thus providing an alternative approach to skeletal cell regeneration. MSCs are quite abundant in tissues, and relatively easy to isolate, characterize and grow in culture as adherent cells [23,61,65,67].

Human MSCs exhibit fibroblastic morphology [67,73,74,75,76], express unique cell surface markers for identification [38,77] and have the unique ability to be guided differentiated in multiple cell types, such as adipocytes, osteoblast and osteoclasts, chondrocytes (cartilage-producing cells), endothelial cells and possibly neuron-like cells (Tsiftsoglou A, unpublished data) (Table 2). In addition, they suppress immune responses, they are anti-inflammatory, express the EPO-R and are responsive to EPO. Interestingly, MSCs have the ability to produce and secrete exosomes (secretome) [38] containing a wealth of various growth factors, cytokines such as angiopoietin, VEGF and several others, that can potentially serve as modern therapeutics for in situ regeneration of damaged tissues observed in several degenerative/metabolic disorders. Therefore, MSCs can be a suitable model system to develop autologous and even allogeneic cell therapy products without provoking severe immunogenicity (see later Section 8 for regenerative medicine).

Studies carried out over the years with stromal BMMSCs have shown that exposure of these cells to EPO prior to transplantation into infarcted heart tissue of animals increased the expression of proangiogenic and survival factors, including HIF1α, angiopoietin, VEGF, VEGFR2, EPO, Bcl-2 and Bcl-xL, following the upregulation of angiogenesis. The MSC-mediated cell therapy of the heart infarct was more beneficial under hypoxic conditions, since it led to CD31^+^ micro-vessel cell formation and enhanced survival of the transplanted cells [32,47]. The more recent study by Noguchi’s group [55] demonstrated the role of endogenous and exogenous EPO on non-erythroid cells such as osteoblasts and adipocytes in transgenic mice with chronic overexpression of EPO and hematocrit, as well as in other mice genetically deficient in EPOR in BM stromal cells. The latter animals lacked EPO–EPO-R-induced signaling and exhibited a reduced ability to form BM osteoblasts and adipocytes. The overall conclusion of this elegant work [55] was that loss of EPO signaling facilitates the production of adipocytes and promotes fat accumulation in the BM at the expense of osteogenesis. Moreover, the same study indicated that endogenous EPO signals regulate bone marrow stromal cell fate and aberrant EPO levels contribute to their impaired differentiation.

As we were quite interested to further explore the angiogenic potential of human MSCs discussed earlier [37] and examine the precise role of EPO in promoting endothelial transdifferentiation, we have employed human dental pulp MSCs (DPSC) and apical papilla mesenchymal stem cells (SCAP) as suitable model systems [59,78,79] to: (i) investigate the angiogenic potential of such cells growing under microenvironmental stress conditions (serum, glucose and/or oxygen deprivation, separately or in combinations), and (ii) explore whether the exposure of SCAP cells to EPO activates the EPO/EPOR signaling pathway, leading to angiogenesis in vitro. Human SCAP cells are a class of quite interesting multipotent MSCs that express several cell surface markers of epithelial, endothelial, neuronal, hematopoietic and embryonic nature [38,59]. By using several methodologies (RT-PCR, flow cytometry, antibody protein microarrays, Western blotting, growth on Matrigel-coated plates to confirm the capillary vessel formation), we provided evidence for endothelial transdifferentiation of SCAP cells growing under different stress conditions [38] or treated with EPO in culture [39].

Furthermore, we assessed the secretome of the SCAP cells, that is their capacity to secrete a large number of growth factors (VEGF, VEGFR2, angiopoietin-1 and others) that can be considered as valuable therapeutics for tissue regeneration. Conditioned medium harvested from cultures of SCAP cells grown under stress, when used in cultures of human HUVEC cells, promoted capillary vessel formation in vitro [38]. Moreover, when SCAP cells were treated with human EPO in culture, they exhibited cell morphology alterations, increased cell longevity and endothelial transdifferentiation, as characterized by the formation of microvascular structures on Matrigel-coated surfaces. Finally, such cells expressed the CD31 marker for endothelial cell maturation [39]. In conclusion, our studies confirmed the potential of dental SCAP cells for angiogenesis and promoted vascularization induced either by stress hypoxic conditions [38] or by EPO [39]. Such findings can be very valuable for the development of tissue-engineered medicinal products (TEPs) for cell therapies aiming to restore damaged or degenerated dental tissue or non-erythroid tissues such as brain tissue.

## 6. EPO: From Tissue Protection to Modulation of the Innate and Adaptive Immune Responses

Evidence now exists to indicate that EPO, in addition to stimulating BM erythropoiesis by binding to the EPO-R homodimeric receptor on erythroid progenitors, also binds to the heterodimeric complex EPO-R on non-erythroid tissues (brain, heart, retina, kidney and others). This heterodimeric complex consists of one chain of EPO-R and a ubiquitous colony-stimulating factor 2 receptor β-chain (CD131) (Figure 2). This heterodimeric receptor has been called the tissue protective receptor (TPR) of EPO [41,80]. Although the interaction of EPO with TPR does not initiate erythropoiesis, it signals biochemical cascades (activation of PI3K, MARK, STAT5 and others), leading to the protection of cells from apoptosis, degeneration and cytotoxicity in general. The binding of EPO with TPR cytoprotects various cell types, but at relatively higher concentrations as compared to the range that stimulates erythropoiesis.

In vitro and in vivo animal studies have demonstrated that EPO through TPR protects the heart from ischemia reperfusion injury (RPI) during myocardial infarction [48,49,81]. However, clinical studies have thus far failed to demonstrate a clear-cut beneficial effect of EPO on the infarct site or on the left ventricular function several weeks after the myocardial infraction. Brain (CNS) cells also produce EPO under hypoxic stress [28,29,51]. EPO stimulates TPR signaling and improves oxygen supply via the revascularization of the ischemic zone, and protects neurons, oligodendrocytes and astrocytes. EPO also improves the long-term neurologic outcome by protecting cells from damage (apoptosis, inflammation, neurotoxicity) and facilitates neuronal regeneration, injury repair and overall survival. The beneficial effects of EPO on neuron protection are under investigation in major neurodegenerative disorders and stroke [51,52]. 

In the retina, EPO also appears to prevent retinopathy via the stimulation of TPR signaling [82]. The beneficial protecting effects of EPO have additionally been observed in the survival of kidney proximal and distal renal tubular cells undergoing stress and injury [83,84]. Moreover, it has been observed that EPO may also protect renal transplants in models of chronic kidney transplant rejection [85]. The functional expression of EPO-R on immune cells that include monocytes, T- and B-lymphocytes, has suggested that EPO may exert beneficial and modulating effects in both the innate and the adaptive immunity arms [30]. Monocytes were found to produce EPO, and their autocrine EPO–EPO-R signaling is involved in the maintenance of immune self-tolerance. Studies with mice and humans have also yielded evidence that EPO has a rather direct inhibitory effect on memory T-cells, while in contrast, it appears to promote the formation and expansion of regulatory T-cells (Tregs) [28]. Moreover, EPO was found to directly activate the TPR in immune cells and suppress their proinflammatory cytokines, thus protecting them from apoptosis. EPO was also found to directly modulate the activation, differentiation and function of immune cells [29].

## 7. EPO Biosimilars

The development of human erythropoietin alpha via recombinant DNA technology as a valuable biopharmaceutical (biologic) medicine, first by Amgen (Epogen^®^) in the late 1980s, was a breakthrough in the treatment of anemia in patients suffering from chronic renal failure (kidney disease) or undergoing cancer chemotherapy [86]. Moreover, epoetin alpha (rhEPO) has been successfully i.v. administered in patients to increase RBCs content and hemoglobin levels and reduce the rate of blood transfusions in adults with moderate anemia or myelodysplastic syndromes (MDS) [87]. Evidence now exists to indicate that EPO directly affects single-cell hematopoietic stem cell differentiation after transplantation, as shown by barcoding tracing analysis [9]. Due to the extensive worldwide demand for epoetins and other effective biologics of the first generation (peptide hormones, growth factors, antibodies and others), a second generation of biologics with very similar primary structures was gradually developed over the past decade [88,89,90,91].

The manufacturing, evaluation and market authorization approval of such biologics, named ‘Biosimilars’, are regulated in Europe and the USA [89,90,91,92]. Biosimilars with similar primary structures, indications and biological properties are manufactured under stringent industrial conditions, assessed biologically in vitro and in vivo via a full-scale comparability study (based on quality, preclinical and clinical evaluation), along with the use of the corresponding reference material (original agent) and then market-authorized and approved [90,91]. Biosimilars exhibit similar pharmacokinetics and pharmacodynamics as the original agent, but often exhibit differences in their immunogenicity [89]. Since EPO acts primarily in the bone marrow to stimulate erythropoiesis and hemoglobin synthesis in several disorders, including anemias associated with chronic kidney failure, Zidovudine treatment of HIV and cancer chemotherapy, the original Epogen^®^ and a number of other epoetin-alpha biosimilars have been used in the clinic [29,85,89]. These include the approved Retacrit, Binocrit, epoetin-alpha Hexal, Procrit, Abseamed and several others under development, as reviewed in detail elsewhere [85,89]. Quality-wise, EPO-alpha biosimilars exhibit similar physicochemical and pharmacological properties, despite their marginal differences in immunogenicity, as mentioned above. The fact that EPO also acts on non-hematopoietic tissues (brain, heart, skeletal muscle, mast cells and others) via the heterodimeric receptor EPO-R/CD131 (TPR) [42,43,50,56,91,93,94] has also “triggered” the development of a new class of EPO-alpha derivatives, including the carbamylated EPO, the B-surface peptide (HBSP) and the Cyclic Helix B-peptide, all under clinical evaluation for use in various degenerative disorders, as discussed earlier [29,85]. 

## 8. Implications of EPO-Induced Differentiation of HSCs and Multipotent MSCs in Regenerative Medicine

The long-term studies with multipotent hematopoietic cells and MSCs over the past years have indicated that regeneration of damaged tissues and even the cure of severe disorders may be possible [31,44,45,53,95,96,97,98,99,100]. BMMSCs as well MSCs of different origins, including dental [101,102,103,104,105,106], have attracted significant attention due to their unique features and properties. These developments, taken together with the generation of induced pluripotent stem cell (iPSC) and animal cloning technologies, offered a fertile ground for the genesis of regenerative medicine (RM) as a new applied Biomedical Science entity. Among the central aims of RM are the rigorous stem cell research, cell phenotype interconversion and the development of innovative gene- and cell-based therapies overall for the potential treatment/cure of degenerative disorders. Growth factors such as human EPO, with its unique abilities to prevent tissue degeneration, induce bone remodeling and promote angiogenic differentiation of MSCs and erythropoiesis in the bone marrow, can be valuable agents in the development and manufacturing of Advanced Therapy Medicinal Products (ATMPs) [107], aiming to promote tissue regeneration and/or treat/cure severe pathologies.

Such pathologies include genetic/metabolic diseases, erythroid tissue homeostatic disorders such as sickle cell anemia (SCA), thalassemia, other anemias, degenerative disorders of bones, skeletal muscles, cartilage-forming tissue and vascular bed and some forms of cancer, all considered as severe unmet medical needs. This can be achieved via the transplantation of autologous and even allogeneic MSCs derived from different tissues to replace, repair and regenerate the damaged tissues. The European Regulatory Framework (Reg. 1394/2007) [107,108] for ATMPs has already market-authorized and approved cell-based ATMPs [108,109] developed from human MSCs for the treatment of bone lesions and cartilage defects attributed to degeneration of chondrocytes and bone osteoblasts. The development of additional ATMPs based on the ability of EPO to promote endothelial transdifferentiation (angiogenesis) of MSCs may form a basis for the treatment of “red foot” type diseases in diabetic patients and other disorders in the conceivable future.

## 9. Conclusions and Future Challenges

Various aspects related to the pleiotropic action of human EPO-alpha, a well-known growth factor and hormone that stimulates erythropoiesis and increases cell survival/protection in several other tissues of the human body, have been presented. EPO continues to surprise us as it goes beyond erythropoiesis as a very valuable agent, not only for the treatment of anemias of different etiology, but also as a survival/protection factor useful for the development of ATMPs derived from human MSCs and/or other tissue stem cells. Its unique ability to bind to its cell surface receptors (the homodimeric EPO-R in bone marrow cells and the heterodimeric EPO-R/CD131 (TPR) in several non-hematopoietic tissues) to promote cell signaling, leading to cell survival and differentiation of MSCs into several cell types (chondrocytes, osteoblasts, endothelial cells, etc.), makes it a very valuable biologic agent and tool for the future development of cell-based therapeutics aiming to treat various tissue degenerative disorders. 

## Figures and Tables

**Table 2:** Human multipotent mesenchymal stem cells (MSCs) derived from various tissues can be differentiated into diverse cell types for regenerative therapies.

Tissue of Origin	Induced to Differentiate into
Bone marrow (BM)	Osteoblast, osteoclasts, stromal cells, pericytes
Umbilical cord blood (UCB)	Adipocytes, chondrocytes, osteoblasts, epithelial cells
Brain	Neuron-like cells (?)
Heart	Cardiomyocytes
Liver	Hepatocytes
Oral cavity (DPSCs, SCAP)	Osteoblast/odontoblast, endothelial cells, neuron-like cells ^(a)^
Kidney	Interstitial tubular cells
Fat	Chondrocytes, skeletal muscle cells (myoblasts)

MSCs are adherent cells with fibroblastic morphology and are able to form colony-forming units-F (CFU-F). Moreover, they are multipotent, as shown above, immunomodulatory and anti-inflammatory, with an enormous ability (secretome) to synthesize and secrete a large number of growth factors, cytokines, chemokines and other molecules [38]. ^(a)^ Tsiftsoglou A., unpublished data. Human dental MSCs can be guided to differentiate into endothelial cells (angiogenic differentiation) by human EPO-alpha [39].

## Data Availability

Not applicable.

## Abbreviations

EPO: erythropoietin; EPO-R: homodimeric erythropoietin receptor; EPO-R-CD131: heterodimeric erythropoietin receptor; TPR: tissue protection receptor; HGF: human growth factor; MCS: mesenchymal stem cells; CNS: central nervous system; IDC: interstitial peritubular cells; HIF: hypoxia-inducing factor; MEP: megakaryocyte erythroid progenitor; JAK2: Janus activation kinase 2; BMMSCs: bone marrow MSCs; MDS: myelodysplastic syndrome; HUVEC: human umbilical vein endothelial cells; EC: endothelial cells; VEGF: vascular endothelial growth factor; DPSC: dental pulp MSCs; SCAP: Apical Papilla MSCs; RM: regenerative medicine; SCA: Sickle cell anemia, ATMPs: Advanced Therapy Medicinal Products; iPSCs: induced pluripotent stem cells.
